# Spatial gene expression profiling identifies prognostic features of residual tumors after neoadjuvant chemotherapy in triple-negative breast cancer

**DOI:** 10.3389/fonc.2025.1638758

**Published:** 2025-08-18

**Authors:** Hye Sung Won, Yong-Seok Kim, Kyung Jin Seo, Sun-Young Jun, Der Sheng Sun, Mihong Choi, Jung-Sook Yoon, Jae Ho Byun

**Affiliations:** ^1^ Department of Internal Medicine, College of Medicine, The Catholic University of Korea, Seoul, Republic of Korea; ^2^ Department of Surgery, College of Medicine, The Catholic University of Korea, Seoul, Republic of Korea; ^3^ Department of Pathology, College of Medicine, The Catholic University of Korea, Seoul, Republic of Korea; ^4^ Clinical Research Laboratory, Uijeongbu St. Mary’s Hospital, College of Medicine, The Catholic University of Korea, Seoul, Republic of Korea

**Keywords:** breast cancer, neoadjuvant chemotherapy, spatial transcriptomics, tumor associated macrophages, recurrence

## Abstract

**Background:**

The standard treatment for early-stage triple-negative breast cancer (TNBC) is neoadjuvant chemotherapy (NAC) followed by surgery, but patients with residual disease have worse outcomes. We investigated genetic alterations related to recurrence using spatial transcriptomic analyses of residual tumors from patients who had and had not relapsed after NAC for early-stage TNBC.

**Methods:**

Thirteen patients who underwent curative resection after NAC for early-stage TNBC, six of whom experienced recurrence, were included. The residual tumor tissues were stained and analyzed using the NanoString GeoMx Digital Spatial Profiling platform. Changes in gene expression were presented as fold changes compared with the control group, and genes were considered to be differentially expressed if they had an absolute value of log2-fold change ≥ 2.0 at a false discovery rate of < 0.05.

**Results:**

On comparing gene expression in residual cancer cells, eight genes (*S100A9, S100A7, CHI3L1, SLPI, SERPINA3, CASP14, URI1*, and *AZGP1*) were found to be significantly upregulated, and 17 (*ACTA2, IGFBP4, BGN, TPM2, MYLK, MMP7, HLA-DPB1, CRISPLD1, COL1A2, OLFM4, KRT14, HLA-DPA1, COL1A1, COL3A1, IFI6, IFI27*, and *A2M*) were significantly downregulated in patients with recurrence. On comparing gene expression in macrophages, six genes (*SLPI, PABPC1, AZGP1, SUPT7L, RPL22*, and *FDCSP*) were found to be significantly upregulated, and *IFI27* was significantly downregulated in patients with recurrence. No genetic alterations with significant differences were found in T cells. No significant change was observed in the density of macrophages between patients with and without recurrence. However, the density of T cells was relatively lower in patients with than in those without recurrence.

**Conclusion:**

We identified some differentially expressed genes relevant to oncogenic signaling and immunosuppressive tumor-associated macrophages. These findings provide novel insights into factors affecting prognosis in patients with residual disease after NAC for early-stage TNBC.

## 1 Introduction

Triple-negative breast cancer (TNBC), which is defined by the lack of expression of estrogen receptor (ER), progesterone receptor (PR), and human epidermal growth factor receptor 2 (HER2), accounts for approximately 15%–20% of all breast cancers and has a dismal prognosis with higher risk of recurrence and poor survival outcomes (1). TNBC is characterized by early relapses within the first 3 years following diagnosis, and metastasis is more likely to occur in the brain and lungs compared to other subtypes. Breast cancer is classified into four distinct molecular subtypes—luminal A, luminal B, HER2-enriched, and basal-like—based on gene expression profiling analysis. The majority of TNBC are classified as a basal-like subtype. In addition, six subtypes by Lehmann et al. and four subtypes by Burstein et al. were proposed as the TNBC molecular subtypes classification (1).

Chemotherapy is the mainstay of treatment for early-stage and advanced TNBC, and several combination regimens, including anthracyclines, taxanes, alkylating agents, and platinum, are used. Neoadjuvant chemotherapy (NAC) has proven efficacy in the treatment of early-stage TNBC. Patients who achieved a pathologic complete response (pCR) after NAC have a better survival outcome than patients with residual invasive disease (1). Recent studies have reported that the addition of immune checkpoint inhibitors (ICIs) to NAC significantly increases the pCR rate in TNBC, regardless of programmed death ligand 1 (PD-L1) status (2). The CREATE-X trial demonstrated a survival benefit with adjuvant capecitabine in patients with residual invasive disease after NAC for TNBC (3), and is therefore recommended as per current guidelines.

Despite these advances in molecular biology and treatment, approximately 30%–40% of patients who have residual disease in early-stage TNBC experience recurrence within 3 years and have a poor prognosis, with a median survival time of less than 2 years. Therefore, there are unmet needs for treatment strategies that effectively reduce recurrence risk in patients with residual TNBC. In addition, achieving negative resection margins is an important factor that affects the risk of local recurrence after breast conserving surgery. Although efforts to achieve a negative margin using intraoperative frozen section analysis should be prioritized, there are many challenges in clinical practice as follows: limited accuracy, time-consuming procedure, technical issues, and lack of resource (4). As the effect of tumor biology on local recurrence was clearly shown in many studies, a deeper understanding of biological features can help overcome these limitations and improve patient outcomes.

In addition to the tumor itself, the tumor microenvironment (TME), which is composed of various immune cells, stromal cells, and extracellular matrix, is recognized as a key factor in tumor progression (5). Among the various immune cells in TME, infiltrating macrophages, which are known as tumor-associated macrophages (TAMs), are a major component of the TNBC TME. Moreover, TAMs are suggested to play critical roles in immune regulation, cancer progression, and drug resistance. Traditionally, they can undergo differentiation into two broad subtypes: M1-like, which are involved in pro-inflammatory responses with antitumor effects, and M2-like, which have protumorigenic and immunosuppressive functions. However, recent studies have reported that TAMs have highly diverse subpopulations depending on the tumor type. The dynamic interactions between tumor cells and these immune cells in TME are essential to cancer cell proliferation and metastasis (6). Therefore, a comprehensive understanding of the underlying cellular and molecular mechanisms of these interactions can contribute to the development of novel drugs and efficient therapeutic strategies.

Spatial transcriptomics is a potent method for studying the interaction between cancer cells and immune cells in TME within their original context. It can simultaneously assess the gene expression of each cell with spatial information. Therefore, they can provide valuable biological insights into the interactions and dynamics between various cells within tumor and studies utilizing this technology has expanded significantly in recent years (7). Chemotherapy affects various intracellular processes in both tumor and immune cells. These intracellular changes after chemotherapy can play a critical role in the subsequent progression of the disease. Accordingly, we aimed to gain insights into potential therapeutic strategies for improving the prognosis of patients without pCR by performing spatial transcriptomic analyses using residual tumors from patients who had and had not relapsed after receiving NAC and adjuvant capecitabine for early-stage TNBC. Here, we identified some differentially expressed genes related to oncogenic signaling and immunosuppressive TAMs in patients with recurrence, suggesting the crucial role of TAMs in patients with residual disease in early-stage TNBC.

## 2 Materials and methods

### 2.1 Patients

Thirteen patients who underwent curative resection for early-stage TNBC in Uijeongbu St. Mary’s Hospital and Incheon St. Mary’s Hospital were included. All patients received NAC but did not achieve pCR and therefore subsequently received adjuvant capecitabine. Among 13 patients, six patients experienced recurrence and seven did not. The following clinicopathological data were collected retrospectively: age, sex, menopausal status, family history of breast cancer, comorbidity, date of breast cancer diagnosis, date of surgery, types of surgery, pathological stage according to the American Joint Committee on Cancer staging system (7th edition), histological grade, lymphovascular invasion, ER status, PR status, HER2 status, Ki-67 labeling index, NAC, adjuvant chemotherapy, adjuvant radiation therapy, recurrence, date of recurrence, survival, date of death, and cause of death. The biospecimens for this study were provided from the Biobank of Uijeongbu St. Mary’s Hospital, the Catholic University of Korea. This study was conducted in accordance with the Declaration of Helsinki and approved by the Institutional Review Board of The Catholic University of Korea, Uijeongbu St. Mary’s Hospital (No. UC2EESE0186). Informed consent was obtained from all subjects involved in the study.

### 2.2 GeoMx digital spatial profiling

We analyzed the gene expression profiles of 13 residual tumor tissues from six patients with recurrence and seven without. The tumor tissues were stained and analyzed using the NanoString GeoMx Digital Spatial Profiling (DSP) platform (Bluewave Bio, Seoul, Korea). Slides were prepared according to the GeoMx DSP instructions. Briefly, 5-μm thick formalin-fixed, paraffin-embedded (FFPE) tissue sections were mounted on a Superfrost Plus Microscope Slides (Fisher Scientific, Pittsburgh, PA, 12-500-15) according to manufacturer’s instructions. The slides were baked at 60 °C for 2.5 hours to enhance tissue adherence. Deparaffinization was performed using Neoclear (1.09843; Sigma-Aldrich, St. Louis, MO, USA), followed by rehydration using a series of ethanol washes (100% and 95%) and phosphate-buffered saline, as described in the manufacturer’s protocol. Antigen retrieval was performed under high temperature and pressure with 1× tris-ethylenediaminetetraacetic acid (pH 9.0) solution. After post-fixation with 10% neutral-buffered formalin solution (Fisher Scientific, Hampton, New Hampshire, USA), the slides were hybridized with GeoMx Human Whole Transcriptome Atlas panel (NanoString Technologies, Seattle, Washington, USA) in humidity chamber at 37°C for overnight, according to the instructions of the GeoMx NGS slide preparation manual. This panel employed DNA-indexed oligonucleotide probes (DSP barcodes) to target 18,677 transcripts. The tissue was then stained using the following antibodies: cytokeratin (pan) antibody (Alexa Fluor® 532 [AE1/AE3]; NanoString Technologies) to identify the epithelial cells, CD68 antibody (Alexa Fluor® 594 [KP1]; Abcam) to identify macrophages, CD3 epsilon antibody (Alexa Fluor® 647 [C3e/1308]; NOVUS) to identify T cells, and SYTO13 antibody (nuclei; NanoString Technologies) to label DNA. After staining with morphology marker, slides were loaded into GeoMx DSP instrument. The exposure time of each antibody was 100 ms for cytokeratin, 300 ms for CD68 and 300 ms for CD3.

**Figure 1:**
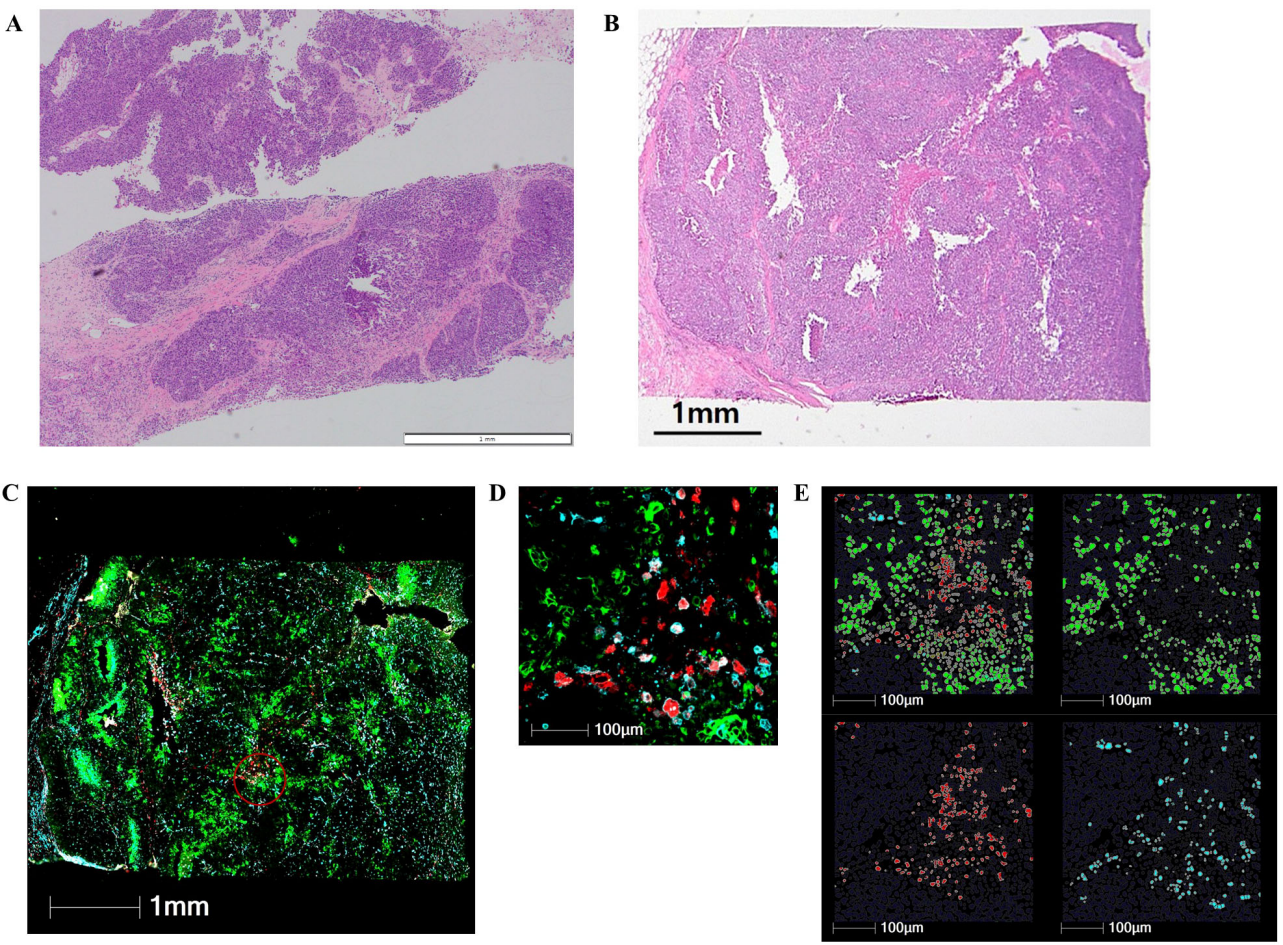
Digital spatial profiling of early-stage triple-negative breast cancer after neoadjuvant chemotherapy. **(A)** Preoperative core needle biopsy of representative case before neoadjuvant chemotherapy (H&E staining, ×40). **(B)** Residual tumor in the surgical specimen after neoadjuvant chemotherapy (H&E staining, ×40). **(C)** Composite digital image of residual tumor annotated using a 600-μm region of interest (red circle). **(D)** Higher magnification image of a region of interest. **(E)** Cell segmentation of pan-cytokeratin (green) for tumor cells, CD3 (blue) for T cells, and CD68 (red) for macrophages.

Regions of interest (ROIs) were carefully selected to exclude areas with histologic evidence of necrosis or edema, while ensuring the inclusion of both tumor and immune cells by blinded pathologists. The two 600µm-diameter ROIs per each sample were included in this analysis. The ROIs were further segmented into areas of illumination based on immunofluorescent markers. The segmentation process classified the cells into three distinct populations: tumor cells (PanCK^+^/CD3^−^/CD68^−^), T cells (CD3^+^), and macrophages (CD68^+^/CD3^−^). The random forest algorithm was applied to ensure accurate cell classification and segmentation based on marker expression and spatial localization (**Figure 1**).

Probes hybridized with RNAs in each ROIs were collected and collected probes were amplified according to the manufacturer’s instructions. Amplified probes were sequenced in NextSeq 2000 system (Illumina, San Diego, CA, USA). Sequencing reads were compiled into FASTQ files and these FASTQ files were converted into digital count files using GeoMxNGSPipeline software.

Quality control was performed using NanoString GeoMx DSP software, according to NanoString’s recommendations. Segments were excluded if they did not meet the following criteria: raw sequencing reads had to be over 1,000 reads and percentage of aligned, trimmed and stitched sequencing had to be over 80%. Probes were excluded if detection rates were below 0.1 or results of Grubbs outlier test were below 20%. Normalization was conducted using the median-of-ratios method (estimateSizeFactors) to correct for sequencing depth and sample composition bias.

### 2.3 Gene set enrichment analysis

Gene set enrichment analysis was performed using the fgsea R package in preranked mode with log2 fold change-based ranking. Gene sets were obtained from MSigDB, including Gene Ontology Biological Process (GO: BP), Hallmark, and Kyoto Encyclopedia of Genes and Genomes (KEGG) pathways with minSize set to 10–15 depending on the category. Adjusted p-value less than 0.05 was considered as the cut-off criterion for analysis.

### 2.4 Statistical analysis

Categorical and continuous variables were compared using the chi-square test and Mann–Whitney *U* test. Differential expression analysis was performed using DESeq2 on the normalized count matrix. Changes in gene expression were presented as fold changes compared with the control group, and expression values were log2-transformed for statistical analysis. Genes were considered to be differentially expressed if they had an absolute value of log2-fold change ≥ 2.0 at a false discovery rate < 0.05. Immune cell deconvolution was performed using Quantiseq (8), applying a Mann–Whitney *U* test to evaluate the relative abundance of immune cell types between groups. Enrichment analysis was conducted on the DESeq2-generated gene expression data, with Fisher’s exact test used to assess the significant activation of gene sets. Relapse-free survival (RFS) was defined as the duration from the date of curative surgery to the date of tumor recurrence or death from any cause. Overall survival (OS) was defined as the duration from the date of diagnosis to the date of death from any cause or the last follow-up. Survival probability was calculated using Kaplan-Meier method to analyze time to event data. All statistical analysis and plot visualizations were performed using R (version 4.3.3).

## 3 Results

### 3.1 Clinicopathological characteristics of the patients

The clinicopathological characteristics of the 13 patients are summarized in **Supplementary Table SI**. The median follow-up time was 43.6 months (range, 31.2–86.4 months). Patients with recurrence had a significantly higher clinical and pathological staging compared to those without recurrence (p = 0.008 and 0.009, respectively). All recurrences occurred within 3 years after curative surgery, and the median interval from the last dose of adjuvant capecitabine to recurrence was 5.8 months (range, 0.1–31.4 months). Three patients had a single site of recurrence (brain, bone, and lymph nodes, respectively). Three patients had recurrence at two or more sites, including the lung, bone, lymph nodes, and skin. The median RFS of patients with recurrence was 11.8 months. All but one of the patients who relapsed died, and the median OS of patients with recurrence was 30.4 months (range, 17.8–57.8 months).

### 3.2 Differentially expressed genes in residual cancer cells between patients with and without recurrence

**Figure 2:**
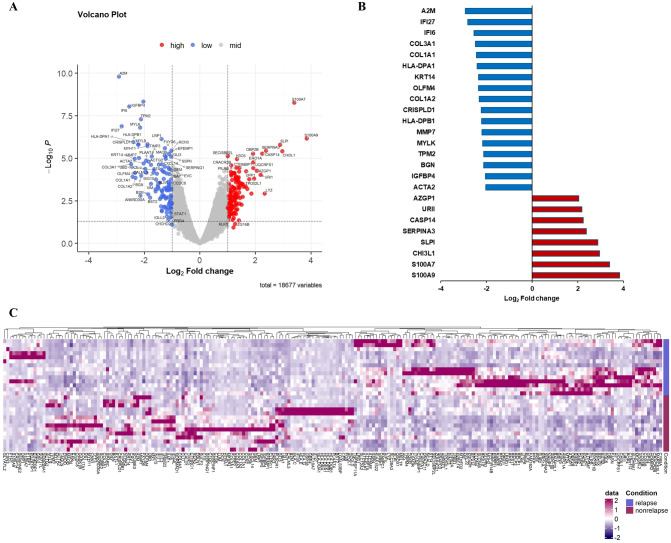
Differential gene expression in tumor cells comparing recurrence and nonrecurrence groups. **(A)** Volcano plot showing differentially expressed genes in tumor cells of patients with compared with those without recurrence. **(B)** Relative mRNA expression of 25 genes with an absolute value of log2-fold change ≥ 2.0 and p < 0.05. **(C)** Heatmap of NanoString gene expression analysis of genes associated with recurrence. The heatmap indicates upregulation (red), downregulation (purple), and mean gene expression (white).

On comparing gene expression in residual cancer cells, we identified 25 differentially expressed genes (DEGs) in patients with compared without recurrence. Eight genes (*S100A9, S100A7, CHI3L1, SLPI, SERPINA3, CASP14, URI1*, and *AZGP1*) were significantly upregulated and 17 (*ACTA2, IGFBP4, BGN, TPM2, MYLK, MMP7, HLA-DPB1, CRISPLD1, COL1A2, OLFM4, KRT14, HLA-DPA1, COL1A1, COL3A1, IFI6, IFI27*, and *A2M*) significantly downregulated in patients with recurrence (**Figure 2**; **Table 1**). Interestingly, the upregulated genes were mainly related to oncogenic signaling pathways and immunosuppressive TME.

**Table 1:** Differentially expressed genes in tumor cells of patients with compared with those without recurrence.

Gene	Full name	Log2FC	Adjusted *P*
Upregulation			
*S100A9*	S100 calcium-binding protein A9	3.843	0.001
*S100A7*	S100 calcium-binding protein A7	3.394	<0.001
*CHI3L1*	Chitinase-3-like protein 1	2.962	0.002
*SLPI*	Secretory leukocyte peptidase inhibitor	2.888	0.001
*SERPINA3*	Serpin family A member 3	2.385	0.002
*CASP14*	Caspase 14	2.245	0.003
*URI1*	Unconventional prefoldin RPB5 interactor 1	2.188	0.014
*AZGP1*	Alpha-2-glycoprotein 1, zinc-binding	2.046	0.011
Downregulation			
*ACTA2*	Actin alpha 2, smooth muscle	−2.029	0.006
*IGFBP4*	Insulin-like growth factor binding protein 4	−2.042	0.001
*BGN*	Biglycan	−2.116	0.015
*TPM2*	Tropomyosin 2	-2.123	<0.001
*MYLK*	Myosin light chain kinase	−2.158	<0.001
*MMP7*	Matrix metallopeptidase 7	−2.199	0.004
*HLA-DPB1*	Major histocompatibility complex, class II, DP beta 1	−2.210	0.001
*CRISPLD1*	Cysteine-rich secretory protein LCCL domain containing 1	−2.219	0.001
*COL1A2*	Collagen type I alpha 2 chain	−2.319	0.018
*OLFM4*	Olfactomedin 4	−2.341	0.012
*KRT14*	Keratin 14	−2.349	0.003
*HLA-DPA1*	Major histocompatibility complex, class II, DP alpha 1	−2.404	0.001
*COL1A1*	Collagen type I alpha 1 chain	−2.431	0.016
*COL3A1*	Collagen type III alpha 1 chain	−2.476	0.007
*IFI6*	Interferon alpha inducible protein 6	−2.549	<0.001
*IFI27*	Interferon alpha inducible protein 27	−2.820	<0.001
*A2M*	Alpha-2-macroglobulin	−2.916	<0.001

S100 proteins are calcium-binding proteins involved in various cellular processes, such as proliferation, differentiation, and inflammation (9). The various functions of each S100 family member have been studied in many cancer types, demonstrating that upregulation of S100 proteins can be associated with tumor growth, metastasis, and drug resistance (9). Previous studies have shown that S100A7 plays a crucial role in tumor growth and metastasis in ER-negative breast cancer cells by activating of extracellular signal-regulated kinase and nuclear factor kappa-light-chain-enhancer of activated B cells signaling. Zhang et al. reported that the high mRNA expression of S100A7 and S100A9 was associated with poor survival in patients with breast cancer (10). Nasser et al. showed that S100A7 overexpression enhanced breast tumor growth and the recruitment of M2 TAMs in a breast cancer mouse model (11). Ma et al. reported that S100A7 expression was related to poor survival outcomes in patients with TNBC treated with NAC and contributed to the progression of TNBC by promoting M2-like macrophage polarization (12). Several studies have demonstrated that S100A9 protein can promote M2 polarization of macrophages and induce immunosuppressive TME by recruiting and accumulating of myeloid-derived suppressor cells in cancer (13). Taken together, S100 protein plays a critical role in oncogenesis by affecting both the tumor and the TME.

Chitinase-3-like protein 1 (CHI3L1) is a secreted glycoprotein that is upregulated in several cancers (14). Recent studies have suggested that CHI3L1 plays a crucial role in tumor immune escape by suppressing antitumor responses. Taifour et al. reported that CHI3L1 expression induced T cell stromal restriction and generated an immunosuppressive TME rich in M2-like macrophages in TNBC (15). They suggested that targeting CHI3L1 can reactivate the antitumor immune response and improve responses to ICIs in cancer.

Secretory leukocyte protease inhibitor (SLPI) is a serine protease inhibitor involved in the modulation of the immunological response and inhibition of protease activities. Recent studies have demonstrated that SLPI expression enhances the metastatic capacity of malignant cells and is associated with poor prognosis in several types of cancer (16). Kozin et al. reported that SLPI secretion directly correlates with lung metastasis in a TNBC mouse model and that higher SLPI expression is associated with worse clinical outcomes in patients with TNBC (17).

Serpin family A member 3 (SERPINA3) is a serine protease inhibitor involved in various biological activities. Its expression is associated with invasion, metastasis, and poor survival outcomes in various cancers (18). Zhang et al. reported that SERPINA3 overexpression promoted proliferation, migration and invasion of TNBC cells and reduced the sensitivity of TNBC cells to cisplatin (19). Another study showed that SERPINA3 was negatively associated with M1-like macrophages and native CD4 T cells, and consequently reduced inflammatory responses and induced immune escape (18).

Caspase 14 (CASP14) belongs to the caspase family and is involved in apoptosis. However, accumulating evidence suggests multiple functions of caspases outside apoptosis in inflammation and tumorigenesis. Handa et al. reported that high CASP14 expression was associated with proliferation, the TNBC phenotype, and cancer stemness. They showed that patients with high CASP14 expression had worse OS and disease-free survival than those with low CASP14 expression in a stages I–III TNBC cohort (20).

Unconventional prefoldin RNA polymerase II subunit 5 interactor 1 (URI1) is important in regulating gene expression and interacts with several transcription factors. It can promote the assembly of multiprotein complexes involved in cell signaling and transcription processes. URI1 has recently been identified as an oncogene in several types of cancer, including ovarian cancer and hepatocellular carcinoma (21).

Zinc-alpha-2-glycoprotein (ZAG/AZGP1), which is called lipid-mobilizing factor, is structurally similar to human leukocyte antigen class I. AZGP1 is a critical protein involved in various physiological processes, including lipid metabolism, immune response, and cancer progression. Hanamura et al. recently reported that AZGP1 was associated with the immune-suppressive phenotype and reduced infiltration of M1-like TAMs in breast cancer, suggesting its role as a regulator of tumor immune response in breast cancer TME (22).

Interferon (IFN) alpha inducible protein 27 (IFI27) and 6 (IFI6) are IFN-stimulated genes (ISGs) that play roles in various biological processes. IFI6 and IFI27 are two related proteins belonging to the FAM14 family that are commonly induced by type I IFNs, and they may regulate innate immune responses to IFNs. Type I IFNs and ISGs can activate innate and adaptive immune cells, including natural killer, CD4-positive T, and cytotoxic CD8-positive T cells (23). Furthermore, IFI27 downregulation is associated with an immunosuppressed microenvironment. Huang et al. reported that high expression of ISGs, including IFI27, was correlated with the increased infiltration of anticancer immune cells, including CD4-positive T cells and pro-inflammatory M1-like macrophages, and improved OS in patients with cancer receiving ICIs (23). Lamsal et al. showed that lower expression of ISGs correlated with a reduced RFS in patients with TNBC and HER2-positive breast cancer (24).

The expression of human leukocyte antigen (HLA; the major histocompatibility complex [MHC] in humans) contributes to the activation of antitumor immunity through interactions with T cell receptors. We found that MHC class II molecules DP alpha1 (HLA-DPA1) and DP beta 1 (HLA-DPB1) were downregulated in patients with recurrence, suggesting suppression of immunity to cancer. MHC I and II molecules can present neoantigens to T cells. Gong et al. reported that HLA I and II expression were associated with the infiltration of antitumor immune cells, including M1-like macrophages and CD8 cytotoxic T, activated CD4 memory T, and natural killer cells, suggesting a positive correlation with immunogenicity (25). In addition, patients with high HLA II expression showed better survival. Schaafsma et al. demonstrated that cancer with high HLA gene expression was associated with an immune-hot TME, and strong positive correlations were found between HLA gene expression and response to ICIs (26).

We also found changes in collagen expression: collagen type I alpha 2 chain (COL1A2), collagen type I alpha 1 chain (COL1A1), and collagen type III alpha 1 chain (COL3A1). Collagen is the main component of the extracellular matrix (ECM) in breast cancer. Many recent studies have indicated both protumorigenic and antitumorigenic roles of collagen during cancer progression. Stewart et al. recently reported that type III collagen played a tumor-suppressive role in human breast cancer. They showed that higher expression of type III collagen was associated with a tumor-restrictive stroma and longer survival in patients with breast cancer, and adding exogenous type III collagen can suppress aggressive breast cancer cell behavior (27). Jansson et al. showed that low type I collagen expression was correlated with a high histological grade, triple-negative subtype, lymph node positivity, and tumor size, indicating that it is a marker of more aggressive breast cancer (28).

Matrix metallopeptidases (MMPs) are a family of endopeptidases that can degrade various proteins in the ECM. They are regulators of cancer invasion and metastasis and play an immunomodulatory role during tumorigenesis and cancer development. Moreover, MMPs may play different roles depending on the types of cancer. Meng et al. performed a comprehensive pan-cancer analysis of the tumorigenic role of MMP7 across human cancers. They confirmed that MMP7 expression had different effects on immune cell infiltration in the TME according to cancer type. Interestingly, MMP7 expression positively correlated with M1-like TAMs but negatively correlated with M2-like TAMs in breast cancer, implying that MMP7 overexpression may cause a shift toward an M1-like phenotype and contribute to better clinical outcomes (29).

Olfactomedin 4 (OLFM4) is a glycoprotein that belongs to the olfactomedin family, which can affect various cellular processes, including proliferation, differentiation, adhesion and innate immunity. Xiong et al. reported that low OLFM4 expression was associated with lymph node and distant metastases. They also reported that patients with low OLFM4 expression had worse OS than those with high OLFM4 expression, indicating that low OLFM4 expression is a poor prognostic factor for patients with TNBC (30).

In addition, we found that genes encoding smooth muscle contractile components (*MYLK, TPM2*, and *ACTA2*) and those associated with ECM (*KRT14, CRISPLD1, BGN*, and *IGFBP4*) were downregulated in patients with recurrence.

### 3.3 Differentially expressed genes in macrophages between patients with and without recurrence

**Figure 3:**
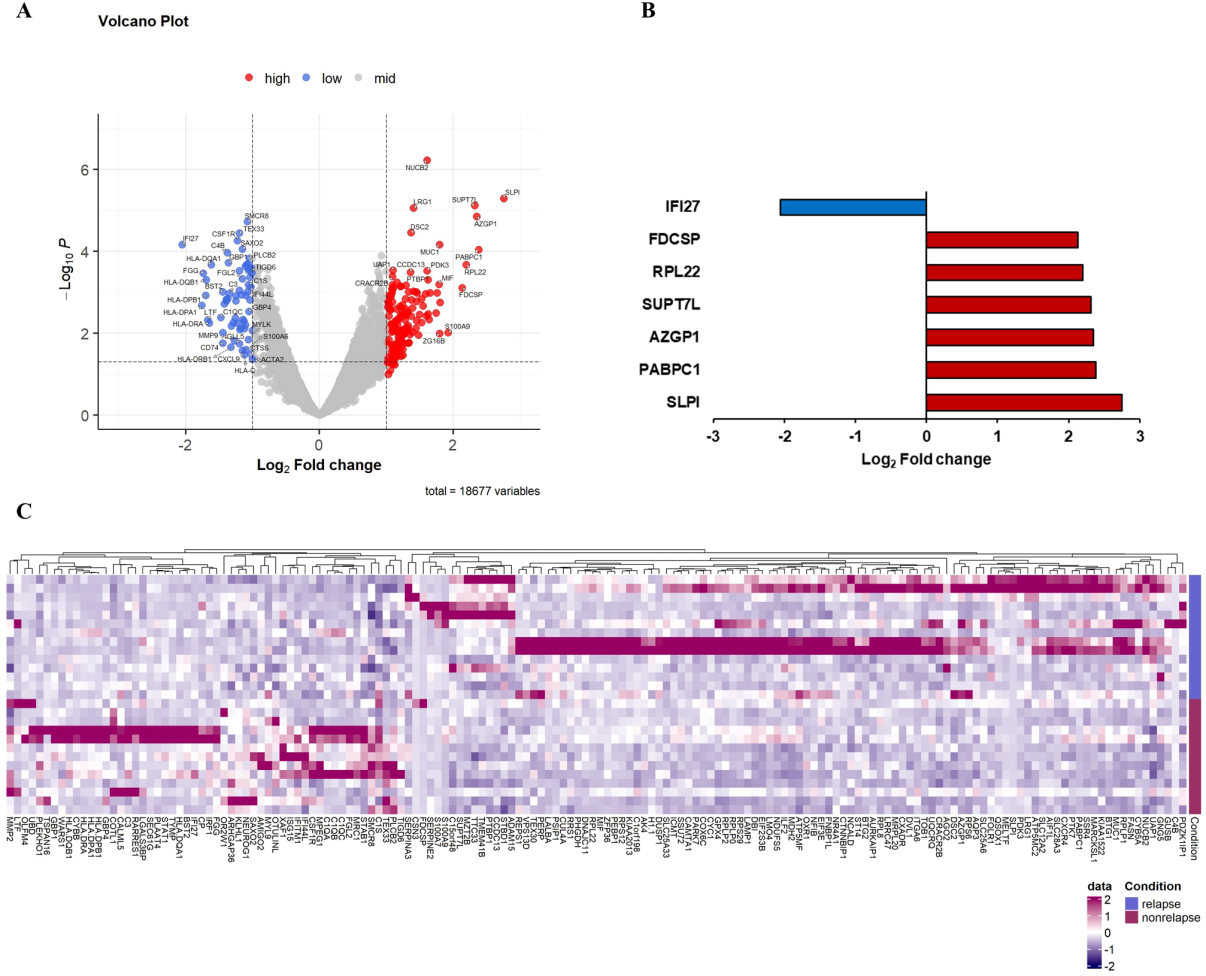
Differential gene expression in macrophages comparing recurrence and nonrecurrence groups. **(A)** Volcano plot showing differentially expressed genes in the macrophages of patients with compared with those without recurrence. **(B)** Relative mRNA expression of seven genes with an absolute value of log2-fold change ≥ 2.0 and p < 0.05. **(C)** Heatmap of NanoString gene expression analysis of genes associated with recurrence. The heatmap indicates upregulation (red), downregulation (purple), and mean gene expression (white).

Macrophages are one of the major components of TME, playing a critical role in cancer progression and metastases. Recent studies have demonstrated that TAMs have highly heterogeneous subpopulations, which can play either protumorigenic or antitumorigenic roles (6). On comparing gene expression in macrophages, we identified seven DEGs in patients with compared with those without recurrence. Six genes (*SLPI, PABPC1, AZGP1, SUPT7L, RPL22, and FDCSP*) were significantly upregulated, and *IFI27* was significantly downregulated in patients with recurrence. Although *CSF1* and *C4B* had significant p-values, their log2-fold changes were found between 1 and 2 (**Figure 3**; **Table 2**).

**Table 2:** Differentially expressed genes in macrophages of patients with compared with those without recurrence.

Gene	Full name	Log2FC	Adjusted *P*
Upregulation			
*SLPI*	Secretory leukocyte peptidase inhibitor	2.754	0.011
*PABPC1*	Poly(A) binding protein cytoplasmic 1	2.379	0.045
*AZGP1*	Alpha-2-glycoprotein 1, zinc-binding	2.352	0.014
*SUPT7L*	SPT7-like, STAGA complex subunit gamma	2.318	0.011
*RPL22*	Ribosomal protein L22	2.197	0.048
*FDCSP*	Follicular dendritic cell secreted protein	2.132	0.049
Downregulation			
*CSF1R*	Colony-stimulating factor 1 receptor	−1.229	0.038
*C4B*	Complement C4B	−1.375	0.048
*IFI27*	Interferon alpha inducible protein 27	−2.051	0.037

Poly(A) binding protein cytoplasmic 1 (PABPC1), which is involved in mRNA regulation and stability, is upregulated in several types of cancer including breast cancer and is associated with a poor prognosis. Shu et al. reported that PABPC1 induced immune evasion and resistance to ICIs in renal cell carcinoma (31). Recently, a genetic biomarker study of acute liver allograft rejection revealed that PABPC1 was negatively correlated with M1-like macrophages and activated natural killer cells, indicating the immune regulatory role of PABPC1 (32).

Follicular dendritic cell secreted protein (FDCSP) is a secreted peptide that modulates various immune responses. Several studies reported that FDCSP is overexpressed in several types of cancer, including breast and ovarian cancers, and contributes to cancer progression and aggressiveness. Recently, Chang et al. reported that high FDCSP expression was associated with poor survival outcomes and was positively correlated with the infiltration of immune cells, including macrophages, in renal cell carcinoma. They also showed that FDCSP expression was positively correlated with PD-1 expression, suggesting that FDCSP expression may affect the effectiveness of ICIs and the immune response to renal cell carcinoma (33).

Colony-stimulating factor 1 receptor (CSF1R) is a receptor tyrosine kinase regulating the proliferation, differentiation, and survival of macrophages. The CSF1R signaling pathway is activated by the binding of CSF1 to the CSF1R. Oshi et al. reported the association between M1-like TAMs or the M1/M2 ratio and cancer biology in breast cancer. They defined M1- and M2-like TAMs by transcriptomic signatures using xCell, as reported by Aran et al. Based on their reference, complement C1q A/B chain, complement C3a receptor 1, CSF1, and CSF1R belong to genes related to M1-like TAMs, and the poly(A) binding protein cytoplasmic 4 (PABPC4) belongs to genes related to M2-like TAMs (34).

Complement activation is associated with the adaptive immune response, and inflammation and may play a role in regulating the T cell response to tumors. Zhang et al. reported that NAC upregulated multiple complement genes, including complement C4B, within the TME, and was associated with a reduction in immune exhaustion gene expression and better survival outcomes in pancreatic cancer (35). These findings suggest that the complement system could affect the prognosis of patients with cancer by modulating the TME.

### 3.4 Functional enrichment analysis of differentially expressed genes in residual cancer cells and macrophages

**Figure 4:**
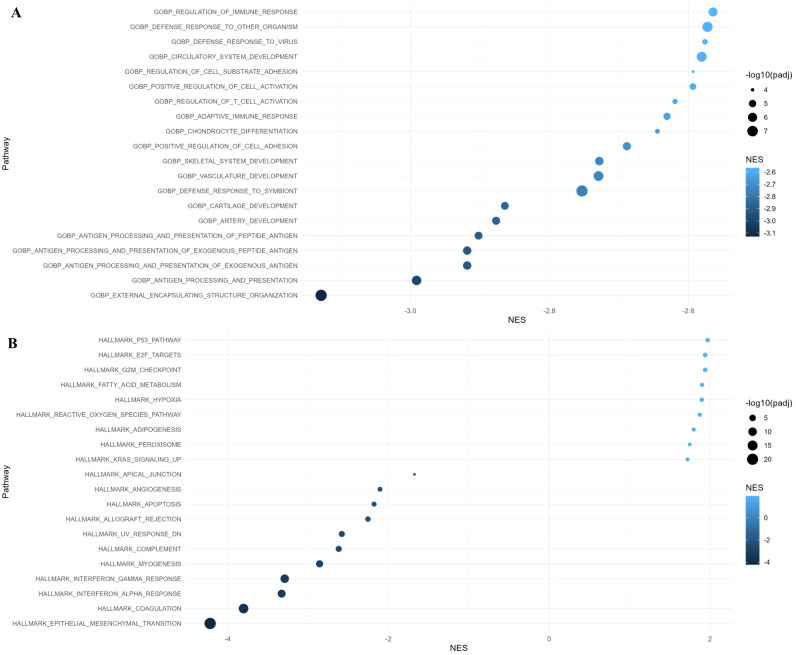
Gene Ontology (GO) and Hallmark pathway analysis of differentially expressed genes with adjusted p-value < 0.05 in residual cancer cells. **(A)** GO Biological Process and **(B)** Hallmark pathway enrichment analysis along with normalized enrichment score (NES).

We performed GO: BP, Hallmark, and KEGG pathway enrichment analysis to gain further insight into their biologic functions of the differentially expressed genes. In the GO: BP analysis, the downregulated genes in residual cancer cells were involved in external encapsulating structure organization, antigen processing and presentation, defense response, positive regulation of cell adhesion, and adaptive immune response. Hallmark gene set enrichment analysis indicated that upregulated genes in residual cancer cells were enriched mainly in the p53 pathway, E2F targets, and G2M checkpoint, while the downregulated genes were enriched in epithelial mesenchymal transition, coagulation, and interferon response (**Figure 4**). KEGG pathways analysis showed that upregulated genes were enriched in translational initiation and mitochondrial complex UCP1 in thermogenesis.

**Figure 5:**
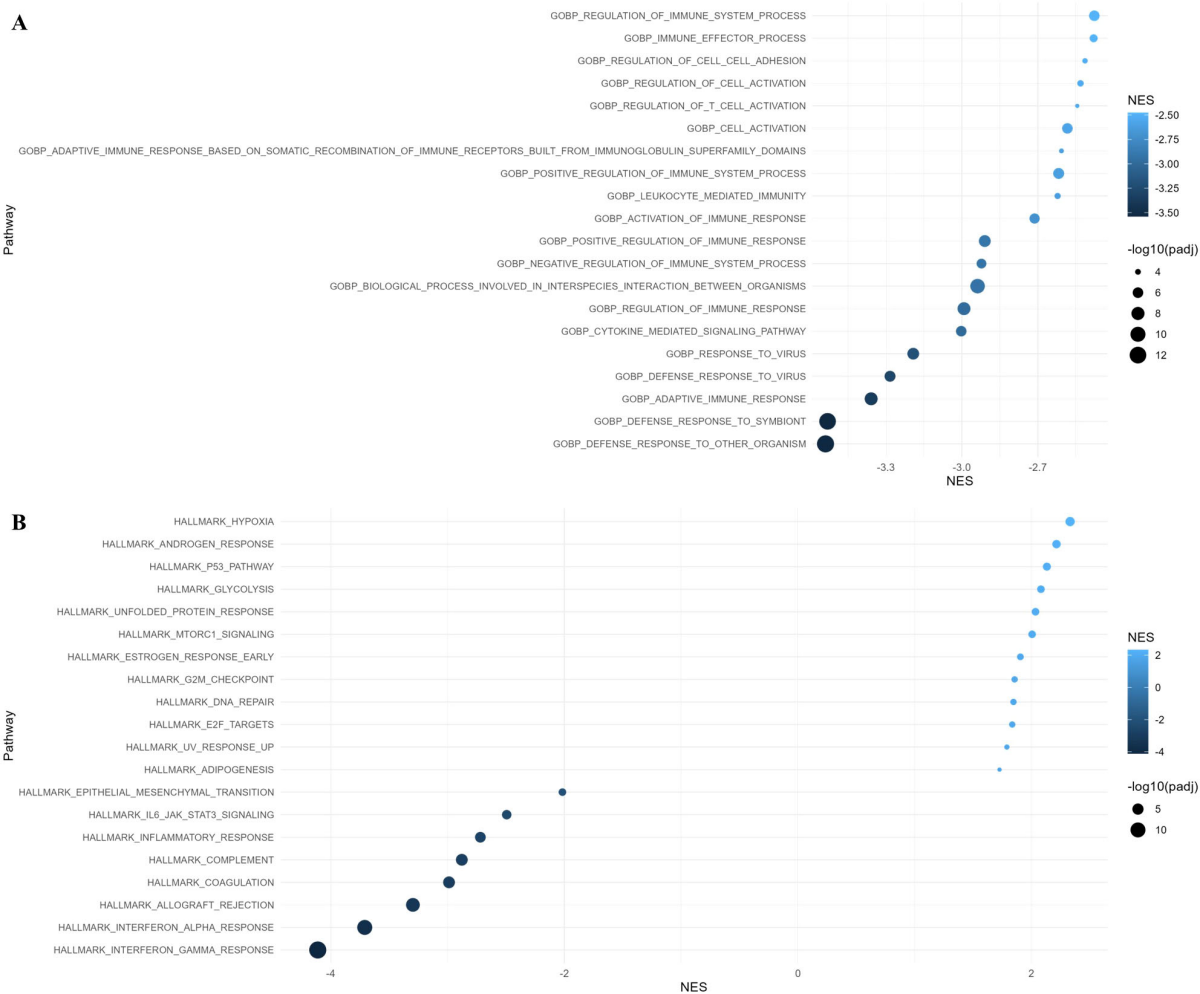
Gene Ontology (GO) and Hallmark pathway analysis of differentially expressed genes with adjusted p-value < 0.05 in macrophages. **(A)** GO Biological Process and **(B)** Hallmark pathway enrichment analysis along with normalized enrichment score (NES).

According to the GO: BP analysis, the downregulated genes in macrophages were enriched in defense response, adaptive immune response, and cytokine mediated signaling pathway. Hallmark gene set enrichment analysis showed that upregulated genes in macrophages were enriched in the hypoxia, androgen response, p53 pathway, and glycolysis, while the downregulated genes were enriched in interferon response, allograft rejection, coagulation, and complement (**Figure 5**).

Taken together, we found several genes with significant alterations in patients who experienced recurrence among those with residual disease after NAC in early-stage TNBC. Based on previous studies on these genes, the activation of specific oncogenic signaling pathways and the induction of immunosuppressive TAMs in residual tumors can contribute to disease recurrence. These findings can be utilized to develop tools for identifying high-risk patients for recurrence among non-pCR patients and further inform decisions on escalating postoperative therapy in these patients. In addition, identification of clinically actionable molecular targets related to recurrence could be leveraged to develop novel therapeutic drugs.

## 4 Discussion

The prominent current trends in cancer treatment are the de-escalation and escalation strategies and biomarker-driven individualized therapies. Prognostic and predictive biomarkers are important factors in identifying patients needing escalation or de-escalation therapy and play a crucial role in new drug development. Traditionally, clinicopathological characteristics, including age at diagnosis, tumor size, lymph node metastasis, histologic grade, and ER, PR, and HER2 status, have been known as important prognostic factors in early-stage breast cancer (1). Recent advances in molecular biology and genetics have revolutionized cancer diagnosis, treatment, and biomarker discovery. Various prognostic and predictive gene signatures have been defined, and the clinical application of genomic analysis has been continuously expanding in recent years. Several multigene assays that evaluate the prognosis or the benefit of adjuvant chemotherapy for hormone receptor (HR)-positive, HER2-negative early-stage breast cancer, are commercially available and widely used in clinical practice (36). The HER2DX assay for early-stage HER2-positive breast cancer also has proven clinical utility improving decision-making and clinical outcomes. The HER2DX genomic test provides three key scores (relapse risk, pCR likelihood, and ERBB2 mRNA scores) by integrating data from 27 genes with clinical factors, tumor size, and nodal status (37). Recently, the TNBC-DX genomic test has been developed and validated to predict the response to NAC and survival outcomes in patients with early-stage TNBC. This genomic test analyzes the 10-gene core immune gene module and the 4-gene tumor cell proliferation signature, while incorporating tumor size and nodal status (38). These recent findings clearly demonstrate the importance of genomic-based biomarkers in optimizing treatment strategies, and this study was initiated in response to recent advances in this field.

NAC has multifaceted effects on both tumor cells and the TME; thus, we investigated genetic alterations associated with relapse by analyzing residual tumor tissues after NAC rather than pretreatment tissues. Accumulating evidence has indicated the relationships between dynamic changes in immune cells of TME after NAC and prognosis in various cancers. Herein, we analyzed not only tumor cells, but also T cells and TAMs using spatial transcriptomics, and identified 25 DEGs in tumor cells and seven in TAMs by comparing patients with and without recurrence. No genetic alterations with significant differences were observed in T cells. Interestingly, genetic alterations of residual tumor cells and TAMs in patients with recurrence showed relevance to oncogenic signaling activation and immunosuppressive TAMs. The most significantly upregulated gene of posttreatment tumor cells in patients with recurrence was *S100A*, which plays a critical role in regulating cell differentiation, proliferation, migration and other biological functions by interacting with various signaling proteins (9). In addition to its role in tumorigenesis, recent studies have revealed that *S100A* may play a pivotal role in immunosuppressive TME and the immunosuppressive activity of macrophages (9). The most significantly downregulated gene of posttreatment tumor cells and macrophages in patients with recurrence was *IFI27*, which plays diverse roles in antitumor immunity (23). Other downregulated genes included *COL1A1, COL3A1*, and *HLA-DP*, indicating the induction of posttreatment immunosuppressed TME in patients with recurrence (25, 27). Therefore, our findings not only provide insights into novel potential therapeutic targets but also demonstrate the importance of reprogramming TAMs to an immunostimulatory state to improve outcomes in patients with residual disease after NAC in early-stage TNBC.

In recent years, numerous studies have investigated TILs, which have been recognized as prognostic and predictive biomarkers for patients with early-stage TNBC, especially in the era of ICIs. Although TAMs comprise a major component of tumor-infiltrating immune cells in the TNBC TME, the importance of TAMs has been relatively underestimated compared with TILs in TNBC. Infiltrated TAMs have been reported to have a higher density in the TNBC TME than in other subtypes of breast cancer (39). TAMs play an essential role in both antitumorigenic and protumorigenic immune responses facilitated by intercellular crosstalk between tumor cells and TAMs (6, 39). TAMs are traditionally divided into the following two subtypes: classical M1-like, which promote pro-inflammatory functions and antitumor immunity, and activated M2-like TAMs, which are important in facilitating tumor metastasis and immunosuppressive activities (39). However, recent single- and bulk-cell genomic studies proposed that TAMs in breast cancer have highly heterogeneous subpopulations (39). Therefore, our study used CD68 as a total macrophage marker instead of M1-like or M2-like markers, such as CD80 and CD163. We found no significant change in the density of CD68-positive macrophages between patients with and without recurrence. However, the density of T cells was relatively lower in patients with than in those without recurrence (**Supplementary Figure S1**). Therefore, our findings suggest that the function of TAMs in the residual tissues of NAC is more important than their number in determining prognosis in early-stage TNBC. Consistent with our findings, Waks et al. suggested a critical role for immunosuppressive macrophage expansion in residual disease by gene expression analysis using paired HR-positive, HER2-negative breast tumors before and after NAC (40). No significant change was observed in the number of CD68-positive macrophages after NAC, but M2-like macrophage-associated genes were upregulated more frequently in the chemotherapy-resistant residual tissues after NAC than M1-like-associated genes (40). This finding suggests the biological role of M2-like macrophages in chemoresistance in HR-positive, HER2-negative breast cancer.

Our findings are consistent with those of previous studies on the immune modulating effect of chemotherapy and suggest that the immune profile of residual tumors after NAC could be considered for personalized targeted therapy to improve clinical outcomes in patients with TNBC. Recent trials have demonstrated that adaptive treatment strategies adopting different adjuvant therapies depending on the response to NAC can overcome drug resistance and improve long-term prognosis (1). Several kinds of TAM-targeting drugs with the following mechanisms are actively being developed (1): promoting phagocytosis of TAMs (2); depletion of M2-like TAMs (3); blocking recruitment of M2-like TAMs (4); reprogramming of TAMs; and (5) suppression of the immunosuppressive TME (41). Although TAMs are an attractive therapeutic target, the following challenges exist in the clinical application of TAM-targeting drugs: heterogeneity of TAMs, different cell–cell interactions, uncovered functions depending on the type of cancer, inconsistent efficacy, and side-effects. Furthermore, TAMs can drive either pro- or antitumorigenic macrophage subpopulations; thus, improving insights into molecular signals controlling the polarization of TAMs can potentially improve therapeutic strategies and the identification of promising new targets. In addition, strategies combining TAM-targeted agents with chemotherapy or immunotherapy could be another option that can induce synergistic therapeutic effects.

The present study has several limitations. First, based on the survival benefit of the KEYNOTE-522 trial, a combination of pembrolizumab with cytotoxic chemotherapy is the current standard of care for NAC in early-stage TNBC (2). However, many patients in Korea receive cytotoxic chemotherapy without ICIs as an NAC because of reimbursement issues; thus, the present study included patients who received cytotoxic chemotherapy without ICIs. In addition, no predictive biomarkers currently exist for neoadjuvant or adjuvant ICIs in early-stage TNBC; thus, our study may help explore these biomarkers in the future. Second, the number of cases included in this analysis was small, and ethnic diversity was not reflected; thus, further validation using an independent external cohort is warranted. Third, as mentioned earlier, we employed CD68 alone as a total macrophage marker, instead of using markers known to distinguish M1-like and M2-like TAMs. Although this considered findings from recent genomic studies on spectrum models of macrophage heterogeneity, moving beyond the traditional binary classification into M1-like and M2-like subtypes (39), it may provide limited information.

In conclusion, we identified potential biomarkers and therapeutic targets that could be relevant to prognosis in patients with residual disease after NAC in early-stage TNBC. Further investigation is needed regarding the functions and biological roles of these genes. Furthermore, our findings suggest that immunosuppressive TAMs may be an essential part of the poor prognosis in these patients. The next step involves validating the impact of these genes on TAMs phenotypes using *in vitro* functional assays, such as macrophage cell-based assays. Further studies are also required to validate the potential of manipulating TAMs phenotype to suppress cancer progression and to elucidate the molecular mechanisms involved in this process. The continued exploration of TAMs and their effects on therapeutic outcomes will be crucial to improve treatment efficacy in patients with TNBC. Although current data on the clinical efficacy of TAM-targeting therapies in patients with breast cancer are limited, these efforts for an in-depth understanding of the various functions of TAMs in TNBC are expected to enable the development of novel potential therapeutic approaches in the future.

## Data Availability

The raw data supporting the conclusions of this article will be made available by the authors, without undue reservation.

## Supplementary material

Supplementary Figure 1 Comparative boxplots of cell density. **(A)** There was no significant change in the density of macrophages between patients with and without recurrence **(B)** The density of T cells was relatively lower in patient with recurrence compared to those without recurrence.
