# Supplementary material for: Spatial gene expression profiling identifies prognostic features of residual tumors after neoadjuvant chemotherapy in triple-negative breast cancer
Source: Front Oncol. 2025 Aug 18;15:1638758. doi: 10.3389/fonc.2025.1638758 (PMC12399621; doi:10.3389/fonc.2025.1638758)
Supplement: Supplementary file 1 [file Table1.docx]

Table S1 Clinicopathological characteristics between patients with and without recurrence

| **Variables** | **Nonrecurrence**  **(n = 7)** | **Recurrence**  **(n = 6)** | **P value** |
| --- | --- | --- | --- |
| Age, Median (range) | 47 (33-60) | 49 (30-66) | 0.606 |
| Menopausal status |  |  | 0.429 |
| Postmenopausal | 2 | 3 |  |
| Presence of family history | 1 | 1 | 0.906 |
| Presence of comorbidity | 2 | 1 | 0.612 |
| Tumor location |  |  | 0.308 |
| Right | 3 | 1 |  |
| Left | 4 | 5 |  |
| Histologic subtypes |  |  | 0.335 |
| Invasive ductal | 6 | 6 |  |
| Others | 1 | 0 |  |
| HER2 status |  |  | 0.429 |
| IHC 0 | 5 | 3 |  |
| IHC +1 or 2+/SISH− | 2 | 3 |  |
| Clinical stage |  |  | 0.008 |
| II | 5 | 0 |  |
| III | 2 | 6 |  |
| Pathological stage |  |  | 0.009 |
| I or II | 7 | 2 |  |
| III | 0 | 4 |  |
| Tumor size |  |  | 0.308 |
| ≤ 2 cm | 3 | 0 |  |
| > 2 cm | 4 | 6 |  |
| Axillary nodal status |  |  | 0.214 |
| 0 | 3 | 1 |  |
| 1-3+ | 4 | 3 |  |
| 4-9+ | 0 | 2 |  |
| Histologic grade |  |  | 0.725 |
| G3 | 3 | 2 |  |
| Presence of LVI | 1 | 1 | 0.906 |
| Ki-67 labelling index |  |  | 0.853 |
| < 20% | 2 | 2 |  |
| Surgery |  |  | 0.048 |
| Lumpectomy | 5 | 1 |  |
| Mastectomy | 2 | 5 |  |
| Type of Neoadjuvant CTx |  |  | 0.450 |
| DE | 0 | 1 |  |
| AC followed by T | 3 | 3 |  |
| TP followed by AC | 4 | 2 |  |

HER2, human epidermal growth factor receptor 2; IHC, immunohistochemistry; SISH, silver-enhanced in situ hybridization; G3, grade 3; LVI, lymphovascular invasion; CTx, chemotherapy; DE, docetaxel, epirubicin; AC, anthracycline, cyclophosphamide; T, taxane; TP, paclitaxel, carboplatin.
