# Supplementary figures and images for: Spatial gene expression profiling identifies prognostic features of residual tumors after neoadjuvant chemotherapy in triple-negative breast cancer
Source: Front Oncol. 2025 Aug 18;15:1638758. doi: 10.3389/fonc.2025.1638758 (PMC12399621; doi:10.3389/fonc.2025.1638758)

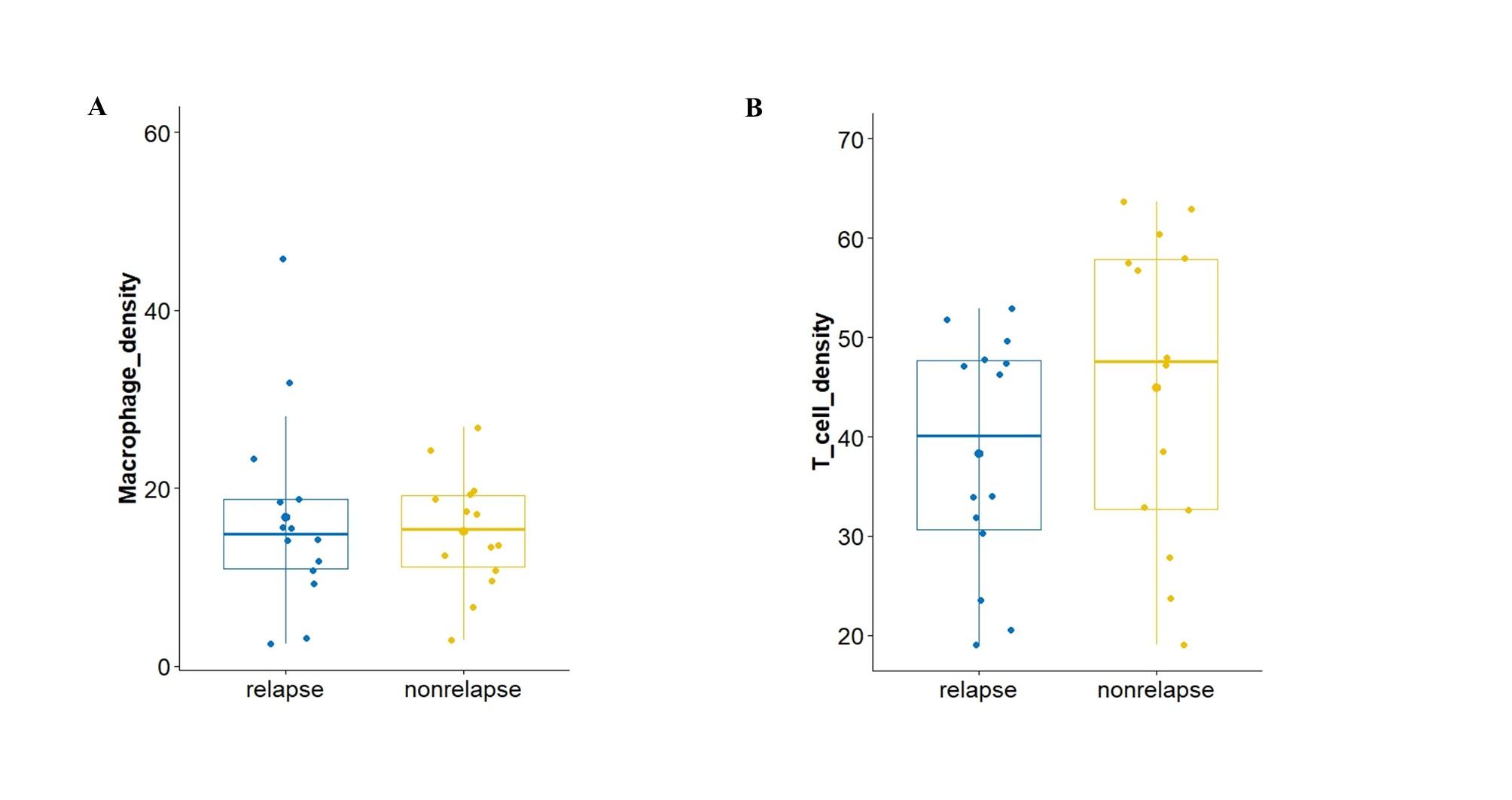

Supplement: Supplementary Figure 1 — Comparative boxplots of cell density. (A) There was no significant change in the density of macrophages between patients with and without recurrence (B) The density of T cells was relatively lower in patient with recurrence compared to those without recurrence. [file Image1.jpeg]
